# Contraceptive use and method preference among HIV-positive women in Amhara region, Ethiopia

**DOI:** 10.1186/s12905-018-0608-y

**Published:** 2018-06-18

**Authors:** Kefyalew Addis Alene, Kendalem Asmare Atalell

**Affiliations:** 1 Institute of Public Health, College of Medicine and Health Sciences, University of Gondar, Gondar, Ethiopia; 2 School of Nursing, College of Medicine and Health Sciences, University of Gondar, Gondar, Ethiopia

**Keywords:** Contraceptive, Family planning, HIV/AIDS, Women, Ethiopia

## Abstract

**Background:**

Providing preferred methods of contraceptive for HIV-positive women and avoiding unintended pregnancy is one of the primary means of preventing mother to child transmission of HIV. This study assessed the prevalence of contraceptive use and method preference among HIV-positive women in Amhara region, Ethiopia.

**Methods:**

A cross-sectional survey was conducted among HIV-positive women in three referral hospitals of Amhara region. Data were collected by interviewing HIV-positive women using a pre-tested and structured questionnaire. A binary logistic regression model was used to identify factors associated with contraceptive use, and odd ratio with 95% confidence interval (CI) was calculated to measure the strength of association.

**Results:**

A total of 803 women living with HIV (with a response rate of 95.4%) were interviewed. The mean age of the study participants was 32.2 years (SD ± 6.2 years). The prevalence of current contraceptive use was 30.3% (95% CI: 27.0–33.7%). The preferred and most commonly used contraceptive methods were injectable (42.8%) and male condom (32.9%). Younger age group (15–24 years) (AOR = 9.67; 95%CI: 3.45, 27.10), one or more number of living children (AOR = 4.01; 95%CI: 2.07, 7.79), HIV diagnosis > 2–4 years (AOR = 2.37; 95%CI: 1.10, 5.08), and having high CD4 count > 500 cell/ul (AOR = 3.25; 95% CI: 1.42, 7.44) were significantly associated with contraceptive use.

**Conclusion:**

The prevalence of contraceptive use among HIV-positive women in Amhara region referral hospitals is low, which suggests a high risk of unintended pregnancy. Injectable and male condoms are the most preferred type of contraceptive methods. Thus, it is better to integrate these contraceptive methods with ART clinic.

**Electronic supplementary material:**

The online version of this article (10.1186/s12905-018-0608-y) contains supplementary material, which is available to authorized users.

## Background

Among HIV infected pregnant women, living in sub-Saharan Africa, around 50–80% of pregnancy is unintended [1, 2]. A study has shown that without any intervention, between 25 and 45% of HIV-positive women living in resource constraint settings transmit HIV to their babies during pregnancy (5–10%), delivery (10–20%), or through breastfeeding (10–20%) [3]. Almost half of the children who acquired HIV infection from their mothers die before their second birthday [3].

Preventing unintended pregnancy among HIV-positive women by providing suitable family planning methods is a critical and cost-effective approach for primary prevention of mother-to-child transmission (PMTCT) of HIV [4]. A cost-effectiveness study has reported that a PMTCT strategy focused on increasing contraception among HIV-positive women could avert 29% more HIV-positive births than prophylactic nevirapine alone, at the same level of expenditure [5]. Preventing unintended pregnancy is a global public health priority for addressing maternal and child health problem in HIV hyper-endemic settings. Addressing the current desperate state of maternal and child health in sub-Saharan Africa is a global public health priority [6]. Central to this agenda is the prevention of unintended pregnancy through increasing access to and use of effective contraception [4]. Although the provision of contraceptive services to HIV-positive women is a critical strategy to prevent unintended pregnancy [7], it is mostly neglected in several settings [8].

The emerging reality of HIV as a manageable chronic disease, with HIV-infected individuals anticipated to live longer and reach their reproductive years, has highlighted the importance of assessing the potential behavioural and biological impacts of ART on contraceptive use and safety [9, 10].

Ethiopia has developed a national guideline on PMTCT of HIV infection. The guideline emphasizes primary prevention of HIV infection and prevention of unintended pregnancies among HIV –infected women [11]. Following the development of this guideline, progress has been observed in the implementation of PMTCT services, and the number of pregnant mothers who receive PMTCT services has increased [12]. However, the overall coverage of PMTCT remains as low as 24% of the expected eligible population [13]. According to the United Nations report, Ethiopia is stated as one of 22 priority countries for eliminating mother-to-child transmission of HIV [14]. The number of pregnant women tested and found to be HIV-positive was 20,755 in 2010–2011; however, only 8365 received antiretroviral drugs (ARVs) [15]. Similarly, only 4945 children born to HIV-infected mothers have received ARV prophylaxis [15]. The reasons for low coverage need to be addressed, and PMTCT uptake needs to be closely linked with efforts to expand coverage of maternal and child health services through the provision of contraceptive [16]. Women with HIV infection, like other women, may wish to plan a pregnancy, limit their family, or avoid unintended pregnancy. However, contraceptive use and method of preference have not been well studied, especially in Amhara region, Ethiopia. Therefore, this study aimed to assess contraceptive use and methods of preference among HIV-positive women.

## Methods

### Study design and setting

A cross-sectional survey was conducted among HIV-positive women in Amhara region, Ethiopia in May 2013. Amhara region is the second largest region in Ethiopia and comprises 23.4% of the total population of the country [17]. The region has five referral hospitals, and the study was conducted in three of these five referral hospitals: Gondar University Referral Hospital, Dessie Referral Hospital, and Debremarkos Referral Hospitals. Two of the hospitals are under the administration of Amhara Regional State Health Bureau, and the other, Gondar University Referral Hospital is a university hospital under the administration of the Federal Ministry of Health.

Debremarkos Referral Hospital is located in East Gojam, 305 km Northwest of Addis Ababa (the capital city of Ethiopia). In the hospital, a total of 8478 people living with HIV (PLHIV) were ever enrolled in the ART clinic, and a total of 1421 women were on ART during the data collection period.

Gondar University Hospital is the oldest hospital in the country and is located in Northwest Ethiopia. It provides health care services for a catchment population of approximately five million people in North Gondar and the adjacent zones. The hospital established HIV care service in 2005 that contains three clinics (i.e. Adult ART clinic, Pediatric ART clinic, and Voluntary Counseling and Testing (VCT) clinic). A total of 10,600 PLHIV were ever enrolled in the ART clinic, and a total of 2562 women were on ART during the data collection period.

Dessie Referral Hospital is located in North Wollo, 400 km Northeast of Addis Ababa. The hospital serves as a referral centre for more than five million people in its catchment area. In the hospital, during the data collection period, a total of 15,985 PLHIV were ever enrolled in ART clinic, and 3015 women were on ART.

### Inclusion and exclusion criteria

Participants were eligible if they were HIV-positive women, aged 15 to 49 years, enrolled in chronic HIV care in the three hospitals (i.e. Gondar, Dessie and Debremarkos referral hospitals), and had at least one visit to the ART unit. HIV-positive women who were pregnant during the data collection period were excluded from the study.

### Sample size and sampling procedures

The sample size for this study was determined using single population proportion formula, by taking the following assumptions: 53.5% contraceptive use among HIV-positive women (from a study conducted at six public hospitals in Addis Ababa, Ethiopia [18]), 5% margin of error, 2 design effect, 10% contingency, and 95% confidence interval. Based on these assumptions, the final sample size was 842. Multistage sampling technique was used to select the study participants. First, three among the five hospitals in Amhara region were selected randomly (i.e. Gondar University Referral Hospital, Debremarkos Referral Hospital and Dessie Referral Hospital). The samples were then proportionally allocated to each hospital. Finally, respondents were selected using systematic random sampling technique.

### Data collection tools and procedures

Data were collected by interviewing HIV-positive women using structured and pre-tested questionnaire. The questioner used in our study was developed for the purpose of this study. It was first prepared in English and then translated into local language (Amharic). The English version of the questionnaire is available as an Additional file 1. Women were interviewed, at the hospitals during their follow up visit, regarding demographics, contraceptive use, HIV and medical history. One supervisor and two data collectors at each hospital, with a total of nine personnel, were participated in the data collection process. To maintain the quality of the data, intensive training was given to the data collectors and supervisors. The data collection process was monitored by the principal investigator. Prior to the main survey, the questioner was pre-tested to assess its clarity and suitability to the study participants. The pre-test was undertaken outside the study sites among 40 HIV-positive women (i.e. 5% of the sample). Participants interviewed in the pre-test were not included in the main study.

### Data processing and analysis

The data were checked for its completeness, entered into Epi Info version 3.5.3, and exported to SPSS version 20.0 software for analysis. Descriptive and summary statistics were calculated. Categorical variables were presented as numbers and percentages, normally distributed continuous variables were presented as mean and standard deviation (SD), and non-normally distributed continuous variables were presented as median and interquartile range (IQR). Bivariate logistic regression models were first performed using demographic and clinical variables. Variables which had a *p* value < 0.2 in the bivariate analysis were then fitted in the final multivariate logistic regression model. Crude and adjusted odds ratios with 95% CI were calculated to measure the strength of association between the dependent and independent variables.

## Results

### Socio-demographic characteristics

A total of 803 HIV-positive women were participated in the interview, with a response rate of 95.4%. The mean age of the women was 32.2 years (SD = 6.2), and the majority of the women (729; 90.8%) were an urban dweller. More than one third (298; 37.1%) were from Gondar University Referral Hospital, 344 (42.8%) were from Dessie Referral Hospital, and 161 (20.0%) were from Debremarkos Referral Hospital. Almost half of the study participants (349; 43.5%) were married. Two-thirds of the women had less than primary education, 282 (35.1%) were housewives, and 219 (27.3%) had a monthly household income less than 300 Ethiopian birr (EBR) (i.e. <$16.6). Amhara (96.8%) and Orthodox Christianity (67.1%) were the dominant ethnicity and religion respectively (Table 1).

**Table 1:** Socio-demographic characteristics of study participants, in Amhara region referral hospitals, Ethiopia, May 2013 (*n* = 803)

Variables	Frequency	Percent
Hospital		
Gondar	298	37.1
Dessie	344	42.8
Debre Markos	161	20.0
Age group (years)		
15–24	67	8.3
25–29	197	24.5
30–34	212	26.4
35–39	202	25.5
40–49	125	15.6
Residence		
Urban	729	90.8
Rural	74	9.2
Marital status		
Single	55	6.8
Married	349	43.5
Divorced	193	24.0
Widowed	184	22.9
Separated	22	2.7
Religion		
Orthodox Christian	539	67.1
Muslim	249	30.8
Protestant	16	2.0
Others	1	0.1
Ethnicity		
Amhara	777	96.8
Tigray	21	2.6
Others	5	0.6
Educational level		
Not able to read and write	218	27.1
Able to read and write	157	19.6
Primary school (1–8)	162	20.2
Secondary School (9–12)	219	27.3
College or University level	47	5.9
Occupation		
Daily labourer	196	24.4
Farmer	59	7.3
Government employee	71	8.8
Housewife	282	35.1
Merchant	126	15.7
Others	69	8.6
Monthly household income (EBR)^a^		
< 300.00	219	27.3
300.00–600.00	191	23.8
601.00–1000.00	232	28.9
> 1000.00	161	20.0

^a^EBR, Ethiopian Birr; Exchanger rate, $ 1 = 18.07 EBR

### HIV infection and reproductive history of women

Half of the study participants (395; 49.2%) had less than three family sizes, one or two previous life birth (405; 50.4%), and one or two surviving children (410; 50.1%). Majority of the women (571; 71.1%) did not give birth after HIV infection, 66 (8.2%) had an HIV-positive child, and two-thirds (549; 68.4%) did not desire future pregnancy. The median CD4 count was 420 cell/ul (IQR = 300–570), and the median time since HIV diagnosis was five years (IQR = 3–6). Almost all of the married women (349; 97.9%) disclosed their HIV status to their husband, and most of their husbands were HIV-positive (73.6%) (Table 2).

**Table 2:** HIV infection and reproductive history of women, in Amhara region referral hospitals, may 2013

Variables	Number	Percent
Family Size		
< 3	395	49.2
3–5	326	40.6
> 5	82	10.2
Previous life birth		
0	127	15.8
1–2	405	50.4
3–4	215	26.8
5+	56	7.0
Number of living children		
0	158	19.7
1–2	410	50.1
3–4	199	24.8
5+	36	4.5
Desires of future pregnancy		
Yes	254	31.6
No	549	68.4
Give birth after HIV infection		
Yes	232	28.9
No	571	71.1
Has or had an HIV-positive child		
Yes	66	8.2
No	677	84.3
I don’t know	60	7.5
Time since HIV diagnosis (years)		
≤ 1 year	86	10.7
2–3 years	301	37.5
≥ 4 years	416	51.8
Current CD4 count		
< 200	78	9.7
200–350	215	26.8
351–500	251	31.3
> 500	259	32.3
HIV status of a husband (*n* = 349)		
Positive	257	73.6
Negative	76	21.8
Unknown	16	4.6
Disclosed HIV status to partner(349)		
Yes	342	97.9
No	7	2.1

### Prevalence of contraceptive use

The overall prevalence of current contraceptive use was 30.3% (95% CI: 27.0–33.7%). The prevalence of contraceptive use among 560 women who do not want to get pregnancy was 31.1% (*n* = 171). Of the 243 women reporting contraceptive use, 36% used contraceptive for 3–5 years. The most commonly used and preferred methods of contraceptive were injectable (42.8%) and male condom (32.9%), followed by the implant (11.1%). Contraceptive use among women before HIV infection was 66.3% and the most commonly used methods were injectable and oral contraceptive (pills). Three hundred and twenty-five women have future need to use a contraceptive. The commonest places where HIV-positive women get contraceptive were at family planning (42.2%), and ART (41.2%) clinic; however, two-thirds of the study participates preferred to get contraceptive at the ART clinic of the hospital. The women were the main decision maker to use contraceptive (Table 3).

**Table 3:** Contraceptive use among HIV-positive women, in Amhara region referral hospitals, May 2013

	Number	Percent
Current use of contraceptive		
Yes	242	30.1
No	561	69.9
Type of currently used contraceptive (*n* = 243)		
Injectable	104	42.8
Male condom	80	32.9
Implants	27	11.1
IUD	15	6.2
Oral contraceptives	12	4.9
Calendar	2	0.8
Lactational Amenorrhea	1	0.4
Others	2	0.8
Time since current contraceptive started (*n* = 242)		
< 6 month	12	4.9
6–12 months	56	23.0
1–2 years	57	23.4
3–5 years	87	35.8
≥ 6 years	31	12.8
Preference method of contraceptive (*n* = 243)		
Yes	237	97.5
No	6	2.5
Place of getting contraceptive in the hospital (*n* = 243)		
FP clinic	103	42.2
ART clinic	100	41.2
Not available in the hospital	36	14.8
Others	4	1.6
Preferred place of getting contraceptive in the hospital (*n* = 243)		
FP clinic	47	19.3
ART clinic	183	75.3
TB clinic	1	0.4
Others	14	5.7
Decision maker to use contraceptive (*n* = 243)		
Women	191	78.6
Partner	36	14.8
Health professional	14	5.8
Others	2	0.8
Contraceptive use prior to HIV infection		
Yes	532	66.3
No	271	33.7
Type of contraceptive used prior to HIV infection		
IUD	8	1.5
Injectable	359	67.5
Norplant	31	5.8
Oral contraceptive/pills	140	26.3
Male condom	11	2.0
Calendar	1	0.2
Future needs for contraceptive		
Yes	325	40.5
No	370	46.1
Not sure	108	13.4

### Factors associated with contraceptive use

In the bivariate analysis, study settings (i.e. hospitals), age of women, marital status, educational level, monthly household income, family size, number of surviving children, giving birth after HIV infection, has or had an HIV-positive child, time since HIV diagnosis and current CD4 count were significantly associated with contraceptive use. However, in the multivariate analysis, the age of women, study settings, marital status, monthly household income, number of surviving children and current CD4 count were remained significantly and independently associated with contraceptive use.

Accordingly, those HIV-positive women who enrolled at Dessie Referral Hospital were less likely to use contraceptive than women who enrolled at Gondar Referral Hospital (AOR = 0.34; 95% CI: 0.19, 0.51). Those women in the age group of 15–24 years (AOR = 9.67; 95%CI: 3.45–27.10), 25–29 years (AOR = 5.86; 95% CI: 2.71, 12.66), 30–34 (AOR = 3.42; 95% CI: 1.64, 7.11) were more likely to use contraceptive as compared to those women with the age group of 40–49 years.

Monthly family income is the other variable which has an association with contraceptive use. Those women whose family monthly incomes 300.00–600.00 EBR were less likely to use contraceptive than women whose monthly income < 300.00EBR (AOR = 0.47; 95%CI: 0.45, 0.87).

The number of living children is also another determinant factor which has an association with contraceptive use. Women who had 1–2 and 3–4 living children were more likely to use contraceptive as compared to women who had no surviving children (AOR = 4.0; 95%CI: 2.07, 7.79) and (AOR =3.11; 95%CI: 1.38, 7.00), respectively.

Duration of HIV diagnosis is also significantly associated with contraceptive use. Those women who have been diagnosed for HIV for a long period (4 years and above) were more likely to use contraceptive as compared to those with infection less than one years (AOR = 2.63; 95%CI: 1.23, 5.61).

Women with a relatively high CD4 count (> 500 cell/ul) were more likely to use contraceptive as compared to low CD4 count (<200cell/ul) (AOR = 3.25; 95% CI: 1.42, 7.44) (Table 4).

**Table 4:** Factors associated with contraceptive use among HIV-positive women in Amhara region referral hospitals, May 2013

Variables	Contraceptive use		COR (95% CI)	AOR(95%CI)
	Yes	No		
Hospital				
Gondar	90	208	1:00	1:00
Dessie	92	252	0.84 (0.59, 1.19)	0.34 (0.19, 0.51)
Debre markos	61	100	1.41 (0.94, 2.11)	1.24 (0.70, 2.20)
Age group (years)				
15–24	22	45	2.90 (1.42, 5.93)	9.67 (3.45, 27.10)
25–29	84	113	4.41 (2.49, 7.84)	5.86 (2.71, 12.66)
30–34	77	135	3.39 (1.91, 6.01)	3.42 (1.64, 7.11)
35–39	42	160	1.56 (0.85, 2.85)	1.99 (0.93, 4.26)
40–49	18	107	1:00	1:00
Marital status				
Married	147	202	1:00	1:00
Single	47	8	8.07 (3.70, 17.59)	0.98 (0.03, 0.26)
Divorced	172	21	0.17 (0.29, 1.72)	0.05 (0.03, 0.10)
Widowed	178	6	0.19 (0.06, 0.59)	0.01 (0.01, 0.04)
Separated	16	6	2.20 (0.66, 7.32)	0.12 (0.04, 0.37)
Religion				
Orthodox Christian	177	362	1:00	
Muslim	62	185	0.68 (0.48, 0.96)	
Protestant	4	13	0.62 (0.20, 1.95)	
Educational level				
Not able to read and write	64	154	0.61 (0.31, 1.17)	
Able to read and write only	39	118	0.48 (0.24, 0.96)	
Primary school (1–8)	46	116	0.58 (0.29, 1.14)	
Secondary School (9–12)	75	144	0.76(0.40, 1.46)	
College or University level	19	28	1:00	
Monthly household income (EBR)^a^				
< 300.00	53	166	1:00	1:00
300.00–600.00	47	144	1.02 (0.65,1.60)	0.47 (0.45, 0.87)
601.00–1000.00	64	168	1.19 (0.78, 1.82)	0.55 (0.30, 1.00)
> 1000.00	79	82	3.01 (1.94, 4.67)	0.57 (0.30, 1.07)
Number of living children				
0	22	136	1:00	1:00
1–2	148	262	3.49 (2.13, 5.72)	4.01 (2.07, 7.79)
3–4	65	134	2.99 (1.74, 5.14)	3.11 (1.38, 7.00)
5+	8	28	1.76 (0.71, 4.36)	2.37 (0.61, 9.20)
Give birth after HIV infection				
Yes	110	122	2.96 (2.15, 4.10)	
No	133	438	1:00	
Has or had an HIV-positive child				
Yes	6	54	1:00	
No	24	42	5.14 (1.92, 13.72)	
I don’t know	213	464	4.13 (1.75, 9.75)	
Time since HIV diagnosis (years)				
≤ 1 year	17	69	1:00	1:00
2–3 years	94	207	1.84 (1.02,3.30)	2.37 (1.10, 5.08)
≥ 4 years	132	284	1.88 (1.06, 3.33)	2.63 (1.23, 5.61)
Current CD4 count				
< 200	13	65	1:00	1:00
200–350	61	154	1.98(1.01,3.85)	1.80 (0.78, 4.15)
351–500	71	180	1.97 (1.02, 3.80)	1.85 (0.81, 4.25)
> 500	98	161	3.04 (1.59,5.80)	3.25 (1.42, 7.44)

^a^EBR, Ethiopian Birr; Exchanger rate, $ 1 = 18.07 EBR

## Discussion

The prevention of unintended pregnancies among HIV infected women is highly dependent on the use of contraceptive. In our study, the overall prevalence of contraceptive use among HIV infected women was 30.3%. This finding is lower than reported prevalence of contraceptive use among reproductive age group women in Ethiopia (36%), and Amhara region (47%) [19]. It is also lower than studies conducted in Brazil (49%), South Africa (53.5%) and Addis Ababa (78%) [20–22]. The lower prevalence of contraceptive use in our study might be explained by the high level of desire for children among the married women, and low levels of sexual desire among the separated, divorced and widowed women. The other reason for the low prevalence of contraceptive use among HIV-positive women could be due to unavailability of the preferred types of contraceptive methods at ART clinics.

Contraceptive methods preferred by women may have implications for both HIV transmission and risks of unintended pregnancy [23, 24]. Although a condom is recommended to prevent HIV transmission to uninfected sexual partners, it is less effective than hormonal contraception and sterilisation at avoiding pregnancy [25]. In our study, among contraceptive users, one third (42.8%) preferred and used male condom exclusively, while a high proportion of women preferred and used an injectable contraceptive.

Majority of women in our study preferred to used injectable contraceptive than condom and oral contraceptive unlike a study conducted in northern Uganda [26], in which the male condom was the most commonly used method (99.4%), followed by the pill (88.3%). This variation might be due to differences in the study participants. In the previous study, the participants were both male and females; whereas, in our study all the participants were female. Previous studies have reported that men are more dominant than women in decision making to use a condom [20, 27]. Women living with HIV infection may feel unable to negotiate condom use with sexual partners for fear of reflectance, domestic violence, loss of economic support, and social isolation [27, 28]. The other reason for not preferring oral contraceptive in our study might be because all the study participants were ART users. Taking both ART and oral contraceptive may increase pill burdens. A survey conducted in South Africa showed a significant difference in oral contraceptive use between ART naïve and ART users [29].

Those HIV-positive women enrolled at ART clinic in Dessie Referral Hospital were 66% less likely to use contraceptive than Gondar Referral Hospital. Even though it needs farther explanation, this difference could be due to a variation in the setup and health professionals in proper counselling on condom use and other contraceptive methods at the ART clinic [30].

Women, less than 35 years of age were more likely to use contraceptive than women 40 years of age. This is because biologically older women will become menopauses and less likely to use contraceptive [21]. The other reason might be older age women have a high chance of separated or divorced from their regular partner so that they may not be sexually active [22, 31]. As a result, they may not use contraceptive. This is supported by our finding that those single, divorced, widowed and separated women are less likely to use contraceptive as compared to married women. This finding also has significant implication for vertical as well as heterosexual transmission of HIV because a large portion of the study participants had a serodiscordant partner (21.8%) and some did not know the HIV status of their partner (4.6%). Besides, 2.1% of those married women had not disclosed their HIV-status to their husband. Women in such situations are less likely to take the necessary care to protect their newborn child from HIV infection [32].

In our study, monthly family income was associated with contraceptive use. Those HIV-positive women with monthly family income 300.00–600.00 EBR were less likely to use contraceptive as compared to those women with family income less than 300.00 EBR. This might be due to the fact that women with a relatively middle income may have a great desire for a child and low intention to use contraceptive [33].

Number of living children was another factor which associated with the use of contraceptive. Similar findings were reported in a study conducted in Uganda [26]. Those women with a high number of living children were more likely to use contraceptive as compared to those women with a low number of living children. This might be explained by the high level of desire for children in low family size women. The strong desire to have children in these women could be the need for future support in their chronic illness [34].

Those women who live with HIV for a long period were more likely to use contraceptive as compared to newly diagnosed women. This is because those women who live with HIV for a long period may have stabilised life in their society and become sexually active [35, 36]. Thus, they may use contraceptive to protect an unplanned pregnancy. In contrast, newly diagnosed women may not be socially stable, and they may not have a desire for sexual intercourse and contraceptive use [35]. The other reason might be those women who live with HIV for a long period could have a better knowledge and understanding of mother to child transmission of HIV than newly diagnosed women; as a result, they may have a desire to use contraceptive [37].

Women with a relatively high CD4 count (> 500 cell/ul) were more likely to use contraceptive as compared to those women having low CD4 count (<200cell/ul). This might be since women with high CD4 count could be relatively healthy and have a desire for sexual intercourse, they might use a contraceptive to prevent unplanned pregnancy.

The study had some limitation. First, due to the cross-sectional nature of the study, the temporal relationship between the explanatory and outcome variable could not be established. Second, considering that the interviews took place at the health facilities, there is a possibility of social desirability bias that may overestimate the prevalence of contraceptive use. However, this was taken care of by recruiting of data collectors out of the hospital staff. Third, this study did not exclude women who were sexually inactive, and it includes only women who are on ART. As a result, we could not see the impact of ART on contraceptive use.

## Conclusion

We found that the prevalence of contraceptive use among HIV-positive women in Amhara region referral hospitals was low. This suggests a high-risk of unintended pregnancy among HIV-positive women. The most preferred contraceptive methods were injectable and male condom. We recommend that these contraceptive methods should be available for HIV-positive women and integrated with the ART clinics.

## Additional file

Additional file 1: The questioner used for the data collection. (DOCX 22 kb)

## Abbreviations

AIDS: Acquired immune deficiency syndrome
AOR: Adjusted odd ratio
ART: Antiretroviral therapy
ARV: Antiretroviral
CI: Confidence interval
EBR: Ethiopian birr
HIV: Human immunodeficiency virus
IQR: Interquartile range
OR: Odd ratio
PLHIV: People living with HIV
PMTCT: Prevention of mother-to-child transmission
SD: Standard deviations
VCT: Voluntary counseling and testing
