# Supplementary material for: Contraceptive use and method preference among HIV-positive women in Amhara region, Ethiopia
Source: BMC Womens Health. 2018 Jun 18;18:97. doi: 10.1186/s12905-018-0608-y (PMC6006570; doi:10.1186/s12905-018-0608-y)
Supplement: Supplementary file 1 — The questioner used for the data collection. (DOCX 22 kb) [file 12905_2018_608_MOESM1_ESM.docx]

**Additional file 1**

The questioner used for the data collection

| **Part I: Socio demographic characteristics of respondents** | | | | | |
| --- | --- | --- | --- | --- | --- |
| S.N | Variables | | Choice | Skip | |
| 101 | Age | | ___________years |  | |
| 102 | Residence | | 1. Rural 2. urban |  | |
| 103 | Marital status | | 1. single 2. married 3. divorced 4. widowed 5. separated |  | |
| 104 | Marriage duration | | _______________years |  | |
| 105 | Religion | | 1. Orthodox 2. Muslim 3. Protestant 4. Other­­­­_______________ |  | |
| 106 | Ethnicity | | 1. Amhara 2. Tigre 3. Others(specify)_______ |  | |
| 107 | Level of education | | 1. Unable to read and write 2. Can read and write only 3. Primary (1-8) 4. Secondary(9-12) 5. College or University |  | |
| 108 | Occupation | | 1. Daily worker 2. Farmer 3. Governmental employee 4. House wife 5. Merchant 6. Others ________________ |  | |
| 109 | husband’s Level of education (if married ) | | 1. Unable to read and write 2. Can read and write only 3. Primary (1-8) 4. Secondary(9-12)   College or University |  | |
| 110 | Family Size | | _____________________ |  | |
| 111 | parity | | _____________________ |  | |
| 112 | No of surviving Children | | _____________________ |  | |
| 113 | No of surviving Males | | _____________________ |  | |
| 114 | Monthly family income | | _____________________Birr |  | |
| Part II: **HIV Related questions** | | | | | |
| 201 | | when you know your HIV status | __________year |  | |
| 202 | | Duration of HAART use | __________year  Don’t start |  | |
| 203 | | Disclosed to partner | 1. Yes 2. No |  | |
| 204 | | Partner’s HIV status | 1. Positive 2. Negative 3. not tested |  | |
| 205 | | Do you have any HIV-infected children | 1. Yes 2. No |  | |
| 206 | | WHO clinical stage | I II III IV |  | |
| 207 | | Recent CD4 cell count | ____________________cell/ul |  | |
| 208 | | Physical functioning | W A B |  | |
| **Part III: pregnancy and family planning related questions** | | | | | |
| 301 | | Are you pregnant now? | 1. yes 2. No 3. unsure | | 2,3🡪3 |
| 302 | | Is it planned? | 1. Yes 2. no | |  |
| 303 | | Would like to have children in the future? | 1. Yes 2. No | |  |
| 304 | | Does your partner desire children | 1. Yes 2. No | |  |
| 305 | | In the last few months have you  Heard about family planning? | 1. Yes 2. No | |  |
| 306 | | What type of method do you heard | _________________________ | |  |
| 307 | | If yes where | 1. Radio 2. Television 3. Newspaper or Magazine 4. Pamphlet/Poster/Leaflets 5. Health institution | |  |
| 308 | | Did you have discussion of family planning with a health worker | 1. Yes 2. No | |  |
| 309 | | Did you have discussion of family planning with a partner | 1. Yes 2. No | |  |
| 310 | | Who is the decision maker to use FP | 1. My self 2. Partner 3. Health worker 4. Others | |  |
| 311 | | knowledge of family planning |  | |  |
| 312 | | perceptions of family planning |  | |  |
| 313 | | Have you ever used anything or tried in any way to delay or avoid getting pregnant | 1. Yes 2. No | |  |
| 314 | | When did you use | Before diagnosed for HIV  After diagnosed for HIV  Both | |  |
| 315 | | Which methods did you use? circle the method ) | 1. Female Sterilization 2. IUD 3. Injectables 4. Implants 5. Pill 6. Male Condom 7. Diaphragm/Foam/Jelly 8. Standard days method 9. Lactational 10. Withdrawal   Other______________ | |  |
| 316 | | Are you currently doing something or using any method to delay or avoid getting pregnant? | 1. Yes 2. No | |  |
| 317 | | Which method are you using? ( write the number from the above list) |  | |  |
| 318 | | For how long did you used this methods | _______years | |  |
| 319 | | Is it your method of choice | 1. Yes 2. No | |  |
| 320 | | Which type of family planning do you like to use? | _______________________ | |  |
| 321 | | Where did you get it? | 1. Hospital 2. Health Center 3. Health Post/HEW 4. Private Clinic 5. Pharmacy 6. Other_______________ | |  |
| 322 | | In this hospital, where do you want to get it | 1. In the family planning clinic 2. In the ART clinic 3. TB clinic 4. Other_______________ | |  |
| 323 | | Do you know of a place where you can obtain a method of family planning? | 1. YES 2. No | |  |
| 324 | | Where is that? | 1. Hospital 2. Health Center 3. Health Post/HEW 4. Private Clinic 5. Pharmacy 6. Other_______________ | |  |
| 325 | | Can you tell me why you are not using a method to prevent pregnancy? | 1. Not Married 2. Not Having Sex 3. Menopausal/Hysterectomy 4. Not Menstruated Since Last Birth 5. Breastfeeding 6. Religious Prohibition 7. Husband/Partner Opposed 8. Lack Of Knowledge 9. Side Effects/Health Concerns 10. Lack Of Access 11. structural barriers 12. Other_____________ | |  |
| 326 | | Do you think you will use a contraceptive method to delay or avoid pregnancy at any time in the future? | 1. Yes 2. No 3. I Don’t know | |  |
| 327 | | Which method do preferred to use | _______________________________ | |  |
